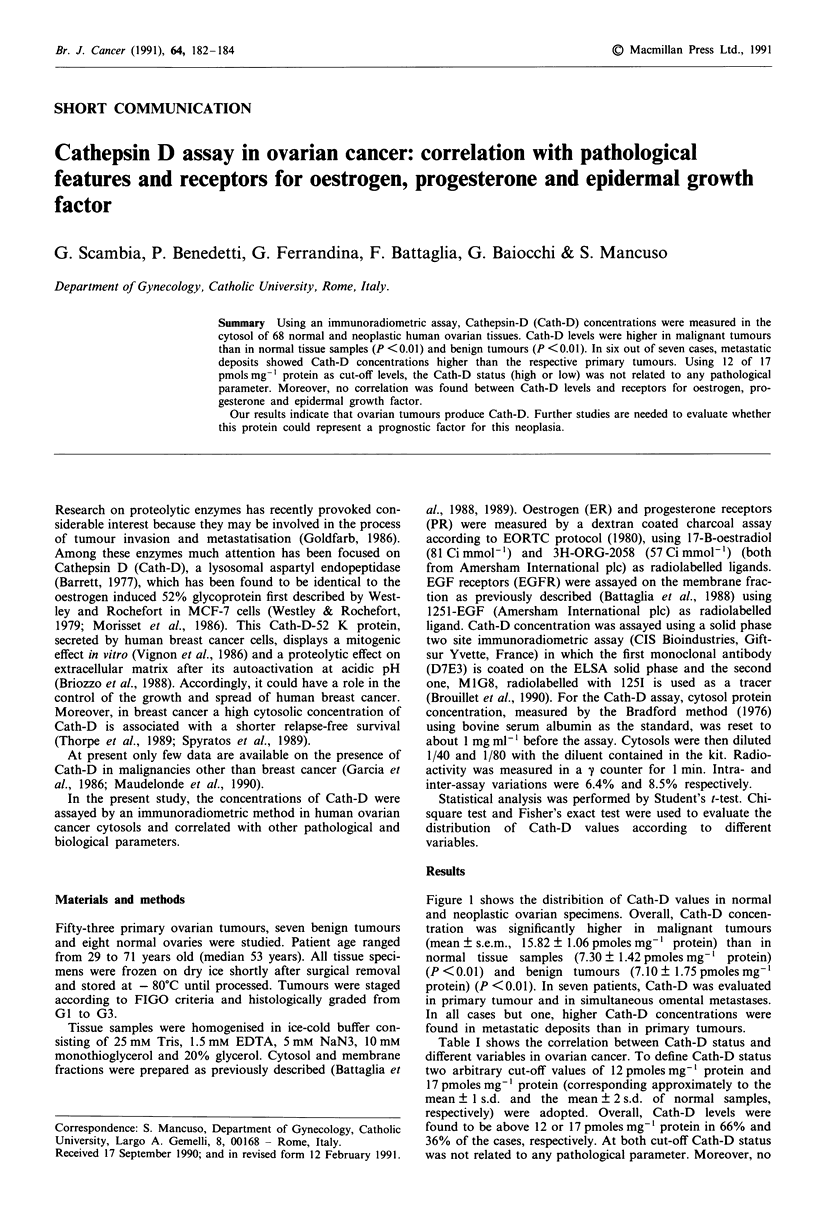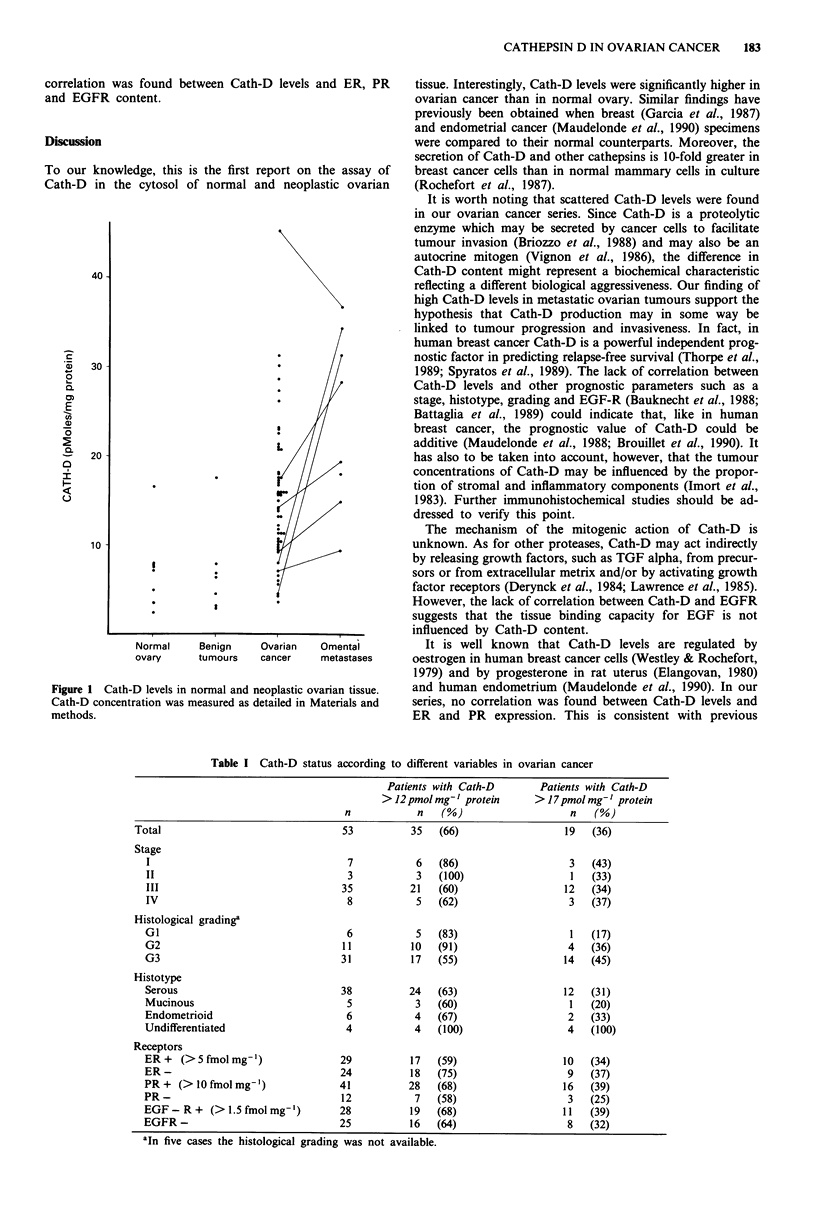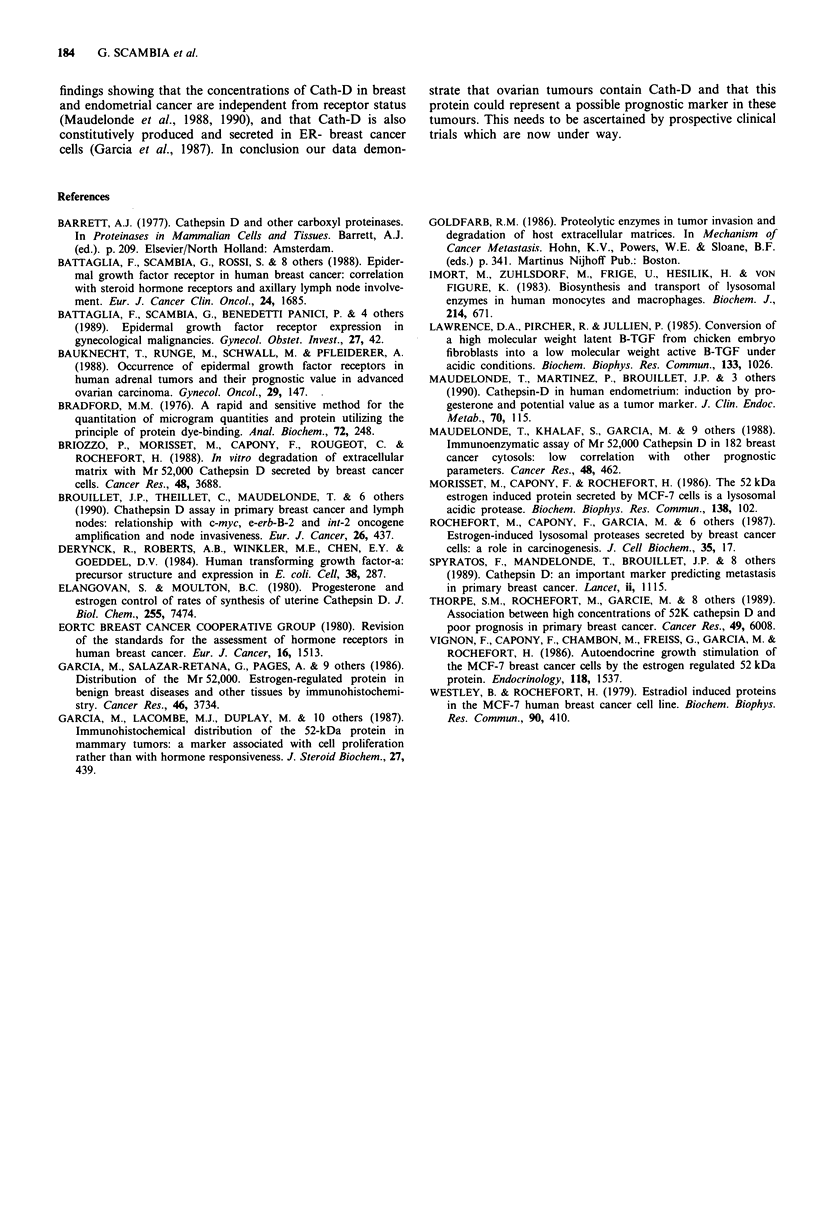# Cathepsin D assay in ovarian cancer: correlation with pathological features and receptors for oestrogen, progesterone and epidermal growth factor.

**DOI:** 10.1038/bjc.1991.266

**Published:** 1991-07

**Authors:** G. Scambia, P. Benedetti, G. Ferrandina, F. Battaglia, G. Baiocchi, S. Mancuso

**Affiliations:** Department of Gynecology, Catholic University, Rome, Italy.

## Abstract

Using an immunoradiometric assay, Cathepsin-D (Cath-D) concentrations were measured in the cytosol of 68 normal and neoplastic human ovarian tissues. Cath-D levels were higher in malignant tumours than in normal tissue samples (P less than 0.01) and benign tumours (P less than 0.01). In six out of seven cases, metastatic deposits showed Cath-D concentrations higher than the respective primary tumours. Using 12 of 17 pmols.mg-1 protein as cut-off levels, the Cath-D status (high or low) was not related to any pathological parameter. Moreover, no correlation was found between Cath-D levels and receptors for oestrogen, progesterone and epidermal growth factor. Our results indicate that ovarian tumours produce Cath-D. Further studies are needed to evaluate whether this protein could represent a prognostic factor for this neoplasia.


Br. J. Cancer (1991), 64, 182-184 © Macmillan Press Ltd., 1991

SHORT COMMUNICATION

### Cathepsin D assay in ovarian cancer: correlation with pathological features and receptors for oestrogen, progesterone and epidermal growth factor

G. Scambia, P. Benedetti, G. Ferrandina, F. Battaglia, G. Baiocchi & S. Mancuso

*Department of Gynecology, Catholic University, Rome, Italy.*

**Summary** Using an immunoradiometric assay, Cathepsin-D (Cath-D) concentrations were measured in the cytosol of 68 normal and neoplastic human ovarian tissues. Cath-D levels were higher in malignant tumours than in normal tissue samples ($P < 0.01$) and benign tumours ($P < 0.01$). In six out of seven cases, metastatic deposits showed Cath-D concentrations higher than the respective primary tumours. Using 12 of 17 pmols mg$^{-1}$ protein as cut-off levels, the Cath-D status (high or low) was not related to any pathological parameter. Moreover, no correlation was found between Cath-D levels and receptors for oestrogen, progesterone and epidermal growth factor.

Our results indicate that ovarian tumours produce Cath-D. Further studies are needed to evaluate whether this protein could represent a prognostic factor for this neoplasia.

Research on proteolytic enzymes has recently provoked considerable interest because they may be involved in the process of tumour invasion and metastatisation (Goldfarb, 1986). Among these enzymes much attention has been focused on Cathepsin D (Cath-D), a lysosomal aspartyl endopeptidase (Barrett, 1977), which has been found to be identical to the oestrogen induced 52% glycoprotein first described by Westley and Rochefort in MCF-7 cells (Westley & Rochefort, 1979; Morisset *et al.*, 1986). This Cath-D-52 K protein, secreted by human breast cancer cells, displays a mitogenic effect *in vitro* (Vignon *et al.*, 1986) and a proteolytic effect on extracellular matrix after its autoactivation at acidic pH (Briozzo *et al.*, 1988). Accordingly, it could have a role in the control of the growth and spread of human breast cancer. Moreover, in breast cancer a high cytosolic concentration of Cath-D is associated with a shorter relapse-free survival (Thorpe *et al.*, 1989; Spyratos *et al.*, 1989).

At present only few data are available on the presence of Cath-D in malignancies other than breast cancer (Garcia *et al.*, 1986; Maudelonde *et al.*, 1990).

In the present study, the concentrations of Cath-D were assayed by an immunoradiometric method in human ovarian cancer cytosols and correlated with other pathological and biological parameters.

### Materials and methods

Fifty-three primary ovarian tumours, seven benign tumours and eight normal ovaries were studied. Patient age ranged from 29 to 71 years old (median 53 years). All tissue specimens were frozen on dry ice shortly after surgical removal and stored at − 80°C until processed. Tumours were staged according to FIGO criteria and histologically graded from G1 to G3.

Tissue samples were homogenised in ice-cold buffer consisting of 25 mM Tris, 1.5 mM EDTA, 5 mM NaN3, 10 mM monothioglycerol and 20% glycerol. Cytosol and membrane fractions were prepared as previously described (Battaglia *et al.*, 1988, 1989). Oestrogen (ER) and progesterone receptors (PR) were measured by a dextran coated charcoal assay according to EORTC protocol (1980), using 17-B-oestradiol (81 Ci mmol$^{-1}$) and 3H-ORG-2058 (57 Ci mmol$^{-1}$) (both from Amersham International plc) as radiolabelled ligands. EGF receptors (EGFR) were assayed on the membrane fraction as previously described (Battaglia *et al.*, 1988) using 125I-EGF (Amersham International plc) as radiolabelled ligand. Cath-D concentration was assayed using a solid phase two site immunoradiometric assay (CIS Bioindustries, Gift-sur Yvette, France) in which the first monoclonal antibody (D7E3) is coated on the ELSA solid phase and the second one, M1G8, radiolabelled with 125I is used as a tracer (Brouillet *et al.*, 1990). For the Cath-D assay, cytosol protein concentration, measured by the Bradford method (1976) using bovine serum albumin as the standard, was reset to about 1 mg ml$^{-1}$ before the assay. Cytosols were then diluted 1/40 and 1/80 with the diluent contained in the kit. Radioactivity was measured in a $\gamma$ counter for 1 min. Intra- and inter-assay variations were 6.4% and 8.5% respectively.

Statistical analysis was performed by Student's *t*-test. Chi-square test and Fisher's exact test were used to evaluate the distribution of Cath-D values according to different variables.

### Results

Figure 1 shows the distribition of Cath-D values in normal and neoplastic ovarian specimens. Overall, Cath-D concentration was significantly higher in malignant tumours (mean ± s.e.m., 15.82 ± 1.06 pmoles mg$^{-1}$ protein) than in normal tissue samples (7.30 ± 1.42 pmoles mg$^{-1}$ protein) ($P < 0.01$) and benign tumours (7.10 ± 1.75 pmoles mg$^{-1}$ protein) ($P < 0.01$). In seven patients, Cath-D was evaluated in primary tumour and in simultaneous omental metastases. In all cases but one, higher Cath-D concentrations were found in metastatic deposits than in primary tumours.

Table I shows the correlation between Cath-D status and different variables in ovarian cancer. To define Cath-D status two arbitrary cut-off values of 12 pmoles mg$^{-1}$ protein and 17 pmoles mg$^{-1}$ protein (corresponding approximately to the mean ± 1 s.d. and the mean ± 2 s.d. of normal samples, respectively) were adopted. Overall, Cath-D levels were found to be above 12 or 17 pmoles mg$^{-1}$ protein in 66% and 36% of the cases, respectively. At both cut-off Cath-D status was not related to any pathological parameter. Moreover, no

Correspondence: S. Mancuso, Department of Gynecology, Catholic University, Largo A. Gemelli, 8, 00168 - Rome, Italy.
Received 17 September 1990; and in revised form 12 February 1991.

correlation was found between Cath-D levels and ER, PR and EGFR content.

### Discussion

To our knowledge, this is the first report on the assay of Cath-D in the cytosol of normal and neoplastic ovarian tissue. Interestingly, Cath-D levels were significantly higher in ovarian cancer than in normal ovary. Similar findings have previously been obtained when breast (Garcia *et al.*, 1987) and endometrial cancer (Maudelonde *et al.*, 1990) specimens were compared to their normal counterparts. Moreover, the secretion of Cath-D and other cathepsins is 10-fold greater in breast cancer cells than in normal mammary cells in culture (Rochefort *et al.*, 1987).

**Figure 1** Cath-D levels in normal and neoplastic ovarian tissue. Cath-D concentration was measured as detailed in Materials and methods.

It is worth noting that scattered Cath-D levels were found in our ovarian cancer series. Since Cath-D is a proteolytic enzyme which may be secreted by cancer cells to facilitate tumour invasion (Briozzo *et al.*, 1988) and may also be an autocrine mitogen (Vignon *et al.*, 1986), the difference in Cath-D content might represent a biochemical characteristic reflecting a different biological aggressiveness. Our finding of high Cath-D levels in metastatic ovarian tumours support the hypothesis that Cath-D production may in some way be linked to tumour progression and invasiveness. In fact, in human breast cancer Cath-D is a powerful independent prognostic factor in predicting relapse-free survival (Thorpe *et al.*, 1989; Spyratos *et al.*, 1989). The lack of correlation between Cath-D levels and other prognostic parameters such as a stage, histotype, grading and EGF-R (Bauknecht *et al.*, 1988; Battaglia *et al.*, 1989) could indicate that, like in human breast cancer, the prognostic value of Cath-D could be additive (Maudelonde *et al.*, 1988; Brouillet *et al.*, 1990). It has also to be taken into account, however, that the tumour concentrations of Cath-D may be influenced by the proportion of stromal and inflammatory components (Imort *et al.*, 1983). Further immunohistochemical studies should be addressed to verify this point.

The mechanism of the mitogenic action of Cath-D is unknown. As for other proteases, Cath-D may act indirectly by releasing growth factors, such as TGF alpha, from precursors or from extracellular metrix and/or by activating growth factor receptors (Derynck *et al.*, 1984; Lawrence *et al.*, 1985). However, the lack of correlation between Cath-D and EGFR suggests that the tissue binding capacity for EGF is not influenced by Cath-D content.

It is well known that Cath-D levels are regulated by oestrogen in human breast cancer cells (Westley & Rochefort, 1979) and by progesterone in rat uterus (Elangovan, 1980) and human endometrium (Maudelonde *et al.*, 1990). In our series, no correlation was found between Cath-D levels and ER and PR expression. This is consistent with previous

**Table I** Cath-D status according to different variables in ovarian cancer

| | | *Patients with Cath-D > 12 pmol mg$^{-1}$ protein* | | *Patients with Cath-D > 17 pmol mg$^{-1}$ protein* | |
|---|---|---|---|---|---|
| | *n* | *n* | *(%)* | *n* | *(%)* |
| Total | 53 | 35 | (66) | 19 | (36) |
| Stage | | | | | |
| I | 7 | 6 | (86) | 3 | (43) |
| II | 3 | 3 | (100) | 1 | (33) |
| III | 35 | 21 | (60) | 12 | (34) |
| IV | 8 | 5 | (62) | 3 | (37) |
| Histological grading[a] | | | | | |
| G1 | 6 | 5 | (83) | 1 | (17) |
| G2 | 11 | 10 | (91) | 4 | (36) |
| G3 | 31 | 17 | (55) | 14 | (45) |
| Histotype | | | | | |
| Serous | 38 | 24 | (63) | 12 | (31) |
| Mucinous | 5 | 3 | (60) | 1 | (20) |
| Endometrioid | 6 | 4 | (67) | 2 | (33) |
| Undifferentiated | 4 | 4 | (100) | 4 | (100) |
| Receptors | | | | | |
| ER + (> 5 fmol mg$^{-1}$) | 29 | 17 | (59) | 10 | (34) |
| ER − | 24 | 18 | (75) | 9 | (37) |
| PR + (> 10 fmol mg$^{-1}$) | 41 | 28 | (68) | 16 | (39) |
| PR − | 12 | 7 | (58) | 3 | (25) |
| EGF − R + (> 1.5 fmol mg$^{-1}$) | 28 | 19 | (68) | 11 | (39) |
| EGFR − | 25 | 16 | (64) | 8 | (32) |

[a]In five cases the histological grading was not available.

findings showing that the concentrations of Cath-D in breast and endometrial cancer are independent from receptor status (Maudelonde et al., 1988, 1990), and that Cath-D is also constitutively produced and secreted in ER- breast cancer cells (Garcia et al., 1987). In conclusion our data demonstrate that ovarian tumours contain Cath-D and that this protein could represent a possible prognostic marker in these tumours. This needs to be ascertained by prospective clinical trials which are now under way.

### References

BARRETT, A.J. (1977). Cathepsin D and other carboxyl proteinases. In *Proteinases in Mammalian Cells and Tissues*. Barrett, A.J. (ed.). p. 209. Elsevier/North Holland: Amsterdam.

BATTAGLIA, F., SCAMBIA, G., ROSSI, S. & 8 others (1988). Epidermal growth factor receptor in human breast cancer: correlation with steroid hormone receptors and axillary lymph node involvement. *Eur. J. Cancer Clin. Oncol.*, **24**, 1685.

BATTAGLIA, F., SCAMBIA, G., BENEDETTI PANICI, P. & 4 others (1989). Epidermal growth factor receptor expression in gynecological malignancies. *Gynecol. Obstet. Invest.*, **27**, 42.

BAUKNECHT, T., RUNGE, M., SCHWALL, M. & PFLEIDERER, A. (1988). Occurrence of epidermal growth factor receptors in human adrenal tumors and their prognostic value in advanced ovarian carcinoma. *Gynecol. Oncol.*, **29**, 147.

BRADFORD, M.M. (1976). A rapid and sensitive method for the quantitation of microgram quantities and protein utilizing the principle of protein dye-binding. *Anal. Biochem.*, **72**, 248.

BRIOZZO, P., MORISSET, M., CAPONY, F., ROUGEOT, C. & ROCHEFORT, H. (1988). *In vitro* degradation of extracellular matrix with Mr 52,000 Cathepsin D secreted by breast cancer cells. *Cancer Res.*, **48**, 3688.

BROUILLET, J.P., THEILLET, C., MAUDELONDE, T. & 6 others (1990). Chathepsin D assay in primary breast cancer and lymph nodes: relationship with c-*myc*, e-*erb*-B-2 and *int*-2 oncogene amplification and node invasiveness. *Eur. J. Cancer*, **26**, 437.

DERYNCK, R., ROBERTS, A.B., WINKLER, M.E., CHEN, E.Y. & GOEDDEL, D.V. (1984). Human transforming growth factor-a: precursor structure and expression in *E. coli*. *Cell*, **38**, 287.

ELANGOVAN, S. & MOULTON, B.C. (1980). Progesterone and estrogen control of rates of synthesis of uterine Cathepsin D. *J. Biol. Chem.*, **255**, 7474.

EORTC BREAST CANCER COOPERATIVE GROUP (1980). Revision of the standards for the assessment of hormone receptors in human breast cancer. *Eur. J. Cancer*, **16**, 1513.

GARCIA, M., SALAZAR-RETANA, G., PAGES, A. & 9 others (1986). Distribution of the Mr 52,000. Estrogen-regulated protein in benign breast diseases and other tissues by immunohistochemistry. *Cancer Res.*, **46**, 3734.

GARCIA, M., LACOMBE, M.J., DUPLAY, M. & 10 others (1987). Immunohistochemical distribution of the 52-kDa protein in mammary tumors: a marker associated with cell proliferation rather than with hormone responsiveness. *J. Steroid Biochem.*, **27**, 439.

GOLDFARB, R.M. (1986). Proteolytic enzymes in tumor invasion and degradation of host extracellular matrices. In *Mechanism of Cancer Metastasis*. Hohn, K.V., Powers, W.E. & Sloane, B.F. (eds.) p. 341. Martinus Nijhoff Pub.: Boston.

IMORT, M., ZUHLSDORF, M., FRIGE, U., HESILIK, H. & VON FIGURE, K. (1983). Biosynthesis and transport of lysosomal enzymes in human monocytes and macrophages. *Biochem. J.*, **214**, 671.

LAWRENCE, D.A., PIRCHER, R. & JULLIEN, P. (1985). Conversion of a high molecular weight latent B-TGF from chicken embryo fibroblasts into a low molecular weight active B-TGF under acidic conditions. *Biochem. Biophys. Res. Commun.*, **133**, 1026.

MAUDELONDE, T., MARTINEZ, P., BROUILLET, J.P. & 3 others (1990). Cathepsin-D in human endometrium: induction by progesterone and potential value as a tumor marker. *J. Clin. Endoc. Metab.*, **70**, 115.

MAUDELONDE, T., KHALAF, S., GARCIA, M. & 9 others (1988). Immunoenzymatic assay of Mr 52,000 Cathepsin D in 182 breast cancer cytosols: low correlation with other prognostic parameters. *Cancer Res.*, **48**, 462.

MORISSET, M., CAPONY, F. & ROCHEFORT, H. (1986). The 52 kDa estrogen induced protein secreted by MCF-7 cells is a lysosomal acidic protease. *Biochem. Biophys. Res. Commun.*, **138**, 102.

ROCHEFORT, M., CAPONY, F., GARCIA, M. & 6 others (1987). Estrogen-induced lysosomal proteases secreted by breast cancer cells: a role in carcinogenesis. *J. Cell Biochem.*, **35**, 17.

SPYRATOS, F., MANDELONDE, T., BROUILLET, J.P. & 8 others (1989). Cathepsin D: an important marker predicting metastasis in primary breast cancer. *Lancet*, **ii**, 1115.

THORPE, S.M., ROCHEFORT, M., GARCIE, M. & 8 others (1989). Association between high concentrations of 52K cathepsin D and poor prognosis in primary breast cancer. *Cancer Res.*, **49**, 6008.

VIGNON, F., CAPONY, F., CHAMBON, M., FREISS, G., GARCIA, M. & ROCHEFORT, H. (1986). Autoendocrine growth stimulation of the MCF-7 breast cancer cells by the estrogen regulated 52 kDa protein. *Endocrinology*, **118**, 1537.

WESTLEY, B. & ROCHEFORT, H. (1979). Estradiol induced proteins in the MCF-7 human breast cancer cell line. *Biochem. Biophys. Res. Commun.*, **90**, 410.